# Suicide in the World

**DOI:** 10.3390/ijerph9030760

**Published:** 2012-03-02

**Authors:** Peeter Värnik

**Affiliations:** 1 Estonian-Swedish Mental Health and Suicidology Institute, Oie 39, Tallinn 11615, Estonia; Email: peeterv@suicidology.ee; 2 Estonian Institute for Population Studies, Tallinn University, Narva mnt 25, Tallinn 10120, Estonia

**Keywords:** suicide, mortality, WHO

## Abstract

*Introduction*: Over the past 20 years the WHO has considerably improved world mortality data. There are still shortcomings but more countries now report data and world-wide estimates are regularly made. *Methods*: Data about mortality have been retrieved from the WHO world database. Worldwide injury mortality estimates for 2008 as well as trends of the suicide rate from 1950 to 2009 were analysed. *Results*: Suicides in the world amount to 782 thousand in 2008 according to the WHO estimate, which is 1.4% of total mortality and 15% of injury mortality. The suicide rate for the world as a whole is estimated at 11.6 per 100,000 inhabitants. The male–female rate ratio of suicide is estimated to be highest in the European Region (4.0) and the lowest in the Eastern Mediterranean region (1.1). Among males the highest suicide rate in the 15–29 age group is in the SE Asian region, in the 45–59 age group in European males and for ages above 60 in the Western Pacific region. Females from SE Asia have a remarkably high suicide rate among 15–29-year-olds and from age 45 in the Western Pacific region. The leading country is currently Lithuania, with a suicide rate of 34.1 per 100,000 inhabitants. Also among males the suicide rate is the highest in Lithuania at 61.2. Among females South Korea with 22.1 is at the top of world suicide rates. *Conclusions*: During the past six decades, according to the WHO Japan, Hungary, and Lithuania have topped the list of world countries by suicide rate, but if the current trends continue South Korea will overtake all others in a few years. The heart of the problem of suicide mortality has shifted from Western Europe to Eastern Europe and now seems to be shifting to Asia. China and India are the biggest contributors to the absolute number of suicides in the world.

## 1. Introduction

It is easy for a well-organised country to measure mortality, including injury mortality, but diagnosing suicide also includes determining the component of intent, which makes it more difficult to have unequivocal statistical data. Given the magnitude suicide mortality and the years of potential life lost, it is certainly worth to learn as much about it as possible trying to overcome the constraints.

Suicide is an individual act but once data are aggregated on a country level the changes from year-to-year are fairly small, there are usually no great fluctuations. It is tempting to state that the best predictor of the suicide rate in the short term is the past suicide rate itself. However, in the longer run large changes can happen and indeed have happened.

There are not that many publications that attempt to analyse the statistics of completed suicides in the whole world. The main reasons for that are probably the deficiencies in the availability and reliability of data. In 1989 Diekstra [1] published suicide rates in 62 countries in connection with analysing socio-demographic trends proposing explanatory theories for international differences. Out of nine top countries only Hungary and Sri Lanka were not from Western Europe. Due to political changes during the past 20 years the number of countries reporting to the European region of the WHO has grown nearly by half. In 1999 Schmidtke *et al*. [2] gave an update about suicide rates in the world and reflected this change. The main improvement in data had been in Europe. In 2002 Bertolote and Fleischmann [3] included improved material from the WHO to make predictions about perspectives of suicide far into the future. They followed it up with several articles and summarised their findings in an article about the global perspective of suicide mortality in 2009 [4]. 

With hindsight it seems that efforts to provide data about injury mortality were stepped up considerably from 2000. Injury mortality includes accidents, suicides, homicides, deaths of undetermined intent and war-related deaths. Reza *et al*. publication [5] and Krug’s report about violence and health in the world [6] documented the new approach. While a lot of data about injury mortality in the world is still lacking or is of questionable quality and according to Rao of the WHO in 2005 only a third of the world’s countries have complete civil registration systems that yield adequate cause-specific mortality data for health policymaking and monitoring [7], the improvements on 20 years ago have been visible—more countries report data and worldwide estimates are regularly made. The aim of the study is to give an overview about the current status of suicide mortality in the world by gender and age, to estimate its proportion among deaths from all causes and injuries, and highlight the most important trends over past six decades.

## 2. Methods

The WHO includes almost all countries that are represented in the United Nations but there are many that do not report the injury mortality statistics. Current data were available for 105 countries, which was just above half of all. Mathers, himself of the WHO, has in 2005 criticised countries for compiling poor vital registration statistics [8]. Still, there was no better worldwide database for analysing suicide and therefore this was chosen as the only source. The WHO uses definitions in accordance to ICD-10 chapter XX to measure injury mortality, including suicide as a separate diagnostic category. While it was rewarding to use national level sources to gain insight into some special cases, it was considered out of the scope of this article to investigate reasons for differences between various sources and possibly also misleading to present different sets of data for the same time periods.

Even so, two datasets were involved in the analysis. The first was prepared by the WHO for 2008 to cover all causes of death and maximise inclusion of countries. Data were estimated according to a similar methodology building on population and all-cause mortality estimates, which was used to get 2002 and 2004 estimates and is described at [9]. The WHO member countries are grouped into six geographic regions: Africa, Americas, Eastern Mediterranean, Europe (including Russia), South-East Asia (including India), and Western Pacific (including China, Japan). Absolute numbers were available in the WHO database. Proportions of suicides among all injury deaths and suicide rates of different age-groups were computed as well as the male-female rate ratios. The second dataset was collected by the WHO from individual countries into the WHO Mortality Database to maximise precision of actually measured suicide mortality data and try to achieve comparability across countries. An overview of latest available data is presented at [10].

**Figure 1:**
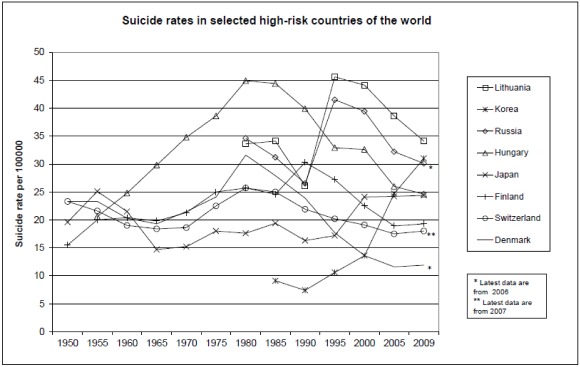
Suicide rates in selected high-risk countries of the world (data were adapted from [11]).

Data by individual countries are presented with 5-year time-intervals starting from 1950 at [11]. The WHO data about gender-specific suicide rates and total numbers, which were available country-by-country, were compiled into an overall table where countries were ranked according to the latest total suicide rate. Male-female suicide rate ratios were computed. Trend analysis was conducted for all countries and the most characteristic development patterns among those with the highest suicide rates over the past six decades were chosen to be presented in Figure 1—two countries from Central Europe, two from North Europe, two from the former Soviet Union, and two from Asia. 

## 3. Results

In May 2011 the WHO published estimates of deaths by cause for the year 2008, which were based on analysis of latest reported national data on mortality cause distributions as known at the end of 2010. The methods used for estimation were briefly explained and the results available online [12]. Table 1 shows the number of suicides by world region against the background of total mortality and injury mortality in particular. Suicides *i.e.*, deaths from self-inflicted intentional injuries amounted to 782 thousand, which was 1.4% of total world mortality ranging from 0.5% in the African region to 1.9% in the South-East Asian region. In all but the European region the amount of road traffic accident casualties prevailed over suicides. Derived from the same database was Table 2, which shows the estimated distribution of suicides in the world by age-groups and gender. Suicide rate for the world as a whole was estimated at 11.6 per 100,000 inhabitants, it was the highest in SE Asia at 15.6 and the lowest in the Eastern Mediterranean region at 5.6. The most clearly outstanding difference between regions was reflected in the male-female rate ratio of suicide—in the European Region it was 4 and in the Americas 3.6 while in the Eastern Mediterranean region it was 1.1 and in the Western Pacific region 1.3. Among males in the 15–29 age-group the suicide rate was the highest in the SE Asian region. The lead was tied in the 30–44 age-group but in the 45–59 age-group European males had the highest suicide rate. For ages above 60 males from the Western Pacific region had the highest suicide rate. Females from SE Asia had a remarkably high suicide rate among 15–29-year-olds and they led also in the next age group. From age 45 the highest female suicide rates were in the Western Pacific region.

Data about latest available suicide rates, which have been measured in individual countries and compiled by the WHO are in Table 3. The leading country was Lithuania with a suicide rate of 34.1 per 100,000 inhabitants. Also among males the suicide rate was the highest in Lithuania at 61.2 but in Russia and Belarus it was also prominent. Among females South Korea with a suicide rate of 22.1 was clearly at the top. Male-female rate ratio was the highest in Puerto Rico (6.6), Slovakia (6.6) and Poland (6.4), while it was the lowest in China (0.9), which is currently the only country where the rate for females is higher than for males.

The countries, which contributed most to the number of suicides in the world, were China, India, Russia, USA, Japan, and South Korea.

Historical data about countries with the highest suicide rates during the past six decades, which are depicted in the Figure 1, illustrate different paths countries have led regarding the development of the suicide trend [11].

Sri Lanka, which has had high suicide rates and a very characteristic suicide trend that best illustrates the importance of the availability of means (pesticides), would have merited inclusion but due to incompleteness of data in the WHO database, it was ultimately omitted.

**Table 1:** Deaths by cause in WHO regions, estimates for 2008.

Cause of Death Population (000)	World		Africa		Americas		Eastern Mediterranean		Europe		South-East Asia		Western Pacific	
	6,737,480		804,865		915,430		580,208		889,170		1,760,486		1,787,321	
	**(000)**	**% total**	**(000)**	**% total**	**(000)**	**% total**	**(000)**	**% total**	**(000)**	**% total**	**(000)**	**% total**	**(000)**	**% total**
**Total Deaths**	**56,888**	**100.0**	**10,125**	**100.0**	**6,170**	**100.0**	**4,198**	**100.0**	**9,223**	**100.0**	**14,498**	**100.0**	**12,674**	**100.0**
**Injuries**	**5,129**	**9.0**	**687**	**6.8**	**594**	**9.6**	**445**	**10.6**	**664**	**7.2**	**1,552**	**10.7**	**1,187**	**9.4**
**Unintentional injuries**	**3,619**	**6.4**	**445**	**4.4**	**355**	**5.8**	**293**	**7.0**	**487**	**5.3**	**1,132**	**7.8**	**908**	**7.2**
Road traffic accidents	1,209	2.1	168	1.7	148	2.4	124	3.0	108	1.2	309	2.1	351	2.8
Poisoning	252	0.4	39	0.4	35	0.6	15	0.4	84	0.9	31	0.2	48	0.4
Falls	510	0.9	19	0.2	48	0.8	24	0.6	66	0.7	211	1.5	142	1.1
Fires	195	0.3	39	0.4	8	0.1	28	0.7	20	0.2	84	0.6	16	0.1
Drowning	306	0.5	42	0.4	20	0.3	22	0.5	27	0.3	96	0.7	98	0.8
Other unintentional injuries	1,146	2.0	136	1.3	96	1.6	79	1.9	181	2.0	401	2.8	252	2.0
**Intentional injuries**	**1,510**	**2.7**	**242**	**2.4**	**239**	**3.9**	**152**	**3.6**	**177**	**1.9**	**420**	**2.9**	**280**	**2.2**
Self-inflicted–Suicide	782	1.4	51	0.5	72	1.2	32	0.8	126	1.4	274	1.9	226	1.8
Violence	535	0.9	162	1.6	157	2.5	22	0.5	46	0.5	102	0.7	47	0.4
War and civil conflict	182	0.3	29	0.3	8	0.1	96	2.3	5	0.1	40	0.3	3	0.0

**Table 2:** Suicide by gender and age groups in WHO regions, estimates for 2008.

WHO region	0–4	5–14	15–29	30–44	45–59	60–69	70–79	80+	Total	Total, male and female
**Males, number of suicides by age groups**										
World	0	5,961	136,532	128,196	117,916	52,222	43,984	21,676	506,487	782,014
Africa	0	724	11,422	9,603	6,939	3,580	2,251	903	35,423	51,332
Americas	0	571	15,171	15,127	13,936	5,309	3,478	2,635	56,227	72,064
Eastern Medit.	0	548	6,753	4,585	2,787	981	896	681	17,230	32,334
Europe	0	509	19,635	25,842	28,615	10,480	9,329	5,070	99,480	125,875
SE Asia	0	2,502	64,548	49,869	33,048	9,965	6,776	2,052	168,761	274,445
Western Pacific	0	1,107	19,002	23,170	32,590	21,907	21,254	10,335	129,366	225,965
**Male suicide rate by age groups**										
World	0.0	0.9	15.3	17.8	23.4	28.2	42.2	60.1	14.9	11.6
Africa	0.0	0.7	10.0	15.1	20.9	30.7	43.0	77.5	8.8	6.4
Americas	0.0	0.7	13.4	15.9	18.9	18.9	22.2	34.1	12.4	7.9
Eastern Medit.	0.0	0.8	7.6	8.1	8.8	9.5	17.0	44.7	5.8	5.6
Europe	0.0	1.0	19.9	26.8	33.6	29.2	37.9	53.2	23.1	14.2
SE Asia	0.0	1.4	25.6	26.8	27.5	25.6	35.8	40.4	18.8	15.6
Western Pacific	0.0	0.8	8.5	10.4	20.4	36.5	61.6	93.5	14.1	12.6
**Females, number of suicides by age groups**										
World	0	5,764	94,959	60,378	48,413	24,791	24,077	17,145	275,527	
Africa	0	354	6,303	2,273	2,775	1,974	1,452	778	15,909	
Americas	0	409	4,311	4,124	4,517	1,378	638	460	15,837	
Eastern Medit.	0	555	8,124	3,443	1,776	410	444	353	15,104	
Europe	0	203	4,001	5,228	7,017	3,464	3,559	2,924	26,395	
SE Asia	0	3,413	59,109	23,793	10,378	5,284	2,635	1,070	105,683	
Western Pacific	0	830	13,112	21,517	21,950	12,282	15,349	11,560	96,599	
**Female suicide rate by age groups**										**Male-female rate ratio**
World	0.0	1.0	11.2	8.6	9.5	12.4	18.7	27.8	8.2	1.8
Africa	0.0	0.3	5.5	3.6	7.7	14.7	22.3	44.5	3.9	2.2
Americas	0.0	0.5	3.9	4.3	5.8	4.4	3.3	3.5	3.4	3.6
Eastern Medit.	0.0	0.9	9.6	6.6	6.0	3.9	8.0	21.1	5.3	1.1
Europe	0.0	0.4	4.2	5.4	7.7	8.1	9.9	14.0	5.8	4.0
SE Asia	0.0	2.0	25.0	13.4	8.9	12.6	11.7	16.6	12.3	1.5
Western Pacific	0.0	0.7	6.3	10.1	14.1	20.5	39.5	64.7	11.1	1.3

**Table 3:** Latest suicide data reported to the WHO.

WHO Region	Country	Year	Suicide rate per 100,000 inhabitants			Number of suicides	Male-female rate ratio
			Male	Female	Total		
Europe	Lithuania	2009	61.3	10.4	34.1	1,138	5.9
Western Pacific	South Korea	2009	39.9	22.1	31.0	15,413	1.8
SE Asia	Sri Lanka	1991	44.6	16.8	31.0	5,347	2.7
Europe	Russian Federation	2006	53.9	9.5	30.1	42,855	5.7
Europe	Belarus	2007	48.7	8.8	27.4	2,663	5.5
Americas	Guyana	2006	39.0	13.4	26.4	202	2.9
Europe	Kazakhstan	2008	43.0	9.4	25.6	4,009	4.6
Europe	Hungary	2009	40.0	10.6	24.6	2,461	3.8
Western Pacific	Japan	2009	36.2	13.2	24.4	30,707	2.7
Europe	Latvia	2009	40.0	8.2	22.9	516	4.9
Europe	Slovenia	2009	34.6	9.4	21.9	447	3.7
Europe	Ukraine	2009	37.8	7.0	21.2	9,716	5.4
Europe	Belgium	2005	28.8	10.3	19.4	2,028	2.8
Europe	Finland	2009	29.0	10.0	19.3	1,032	2.9
Europe	Serbia	2009	28.1	10.0	18.8	1,376	2.8
Europe	Estonia	2008	30.6	7.3	18.1	242	4.2
Europe	Switzerland	2007	24.8	11.4	18.0	1,360	2.2
Europe	Croatia	2009	28.9	7.5	17.8	790	3.9
Europe	Moldova	2008	30.1	5.6	17.4	620	5.4
Europe	France	2007	24.7	8.5	16.3	10,122	2.9
Americas	Uruguay	2004	26.0	6.3	15.8	526	4.1
Europe	Austria	2009	23.8	7.1	15.2	1,273	3.4
Europe	Poland	2008	26.4	4.1	14.9	5,681	6.4
Western Pacific	Hong Kong	2009	19.0	10.7	14.6	1,024	1.8
Americas	Suriname	2005	23.9	4.8	14.4	72	5.0
Europe	Czech Republic	2009	23.9	4.4	14.0	1,464	5.4
Western Pacific	China, selected areas (estimated total)	1999	13.0	14.8	13.9	16,836(200,000)	0.9
Europe	Sweden	2008	18.7	6.8	12.7	1,170	2.8
Europe	Slovakia	2005	22.3	3.4	12.6	679	6.6
Americas	Cuba	2008	19.0	5.5	12.3	1,376	3.5
Europe	Bulgaria	2008	18.8	6.2	12.3	939	3.0
Europe	Romania	2009	21.0	3.5	12.0	2,286	6.0
Europe	Norway	2009	17.3	6.5	11.9	573	2.7
Europe	Iceland	2008	16.5	7.0	11.9	38	2.4
Europe	Denmark	2006	17.5	6.4	11.9	647	2.7
Europe	Germany	2006	17.9	6.0	11.9	9,765	3.0
Europe	Ireland	2009	19.0	4.7	11.8	527	4.0
Europe	Bosnia and Herzegovina	1991	20.3	3.3	11.8	531	6.2
Western Pacific	New Zealand	2007	18.1	5.5	11.7	493	3.3
Americas	Canada	2004	17.3	5.4	11.3	3,613	3.2
Americas	Chile	2007	18.2	4.2	11.1	1,848	4.3
Americas	United States of America	2005	17.7	4.5	11.0	32,559	3.9
Americas	Trinidad and Tobago	2006	17.9	3.8	10.7	141	4.7
SE Asia	India	2009	13.0	7.8	10.5	127,151	1.7
Western Pacific	Singapore	2006	12.9	7.7	10.3	372	1.7
Europe	Portugal	2009	15.6	4.0	9.6	1,025	3.9
Europe	Luxembourg	2008	16.1	3.2	9.6	47	5.0
Europe	Netherlands	2009	13.1	5.5	9.3	1,525	2.4
Europe	Kyrgyzstan	2009	14.1	3.6	8.8	458	3.9
Europe	Turkmenistan	1998	13.8	3.5	8.6	406	3.9
Western Pacific	Australia	2006	12.8	3.6	8.2	1,673	3.6
Americas	El Salvador	2008	12.9	3.6	8.0	490	3.6
Africa	Zimbabwe	1990	10.6	5.2	7.9	768	2.0
SE Asia	Thailand	2002	12.0	3.8	7.8	4,905	3.2
Americas	Argentina	2008	12.6	3.0	7.7	3,068	4.2
Europe	Spain	2008	11.9	3.4	7.6	3,457	3.5
Americas	Puerto Rico	2005	13.2	2.0	7.4	288	6.6
Americas	Ecuador	2009	10.5	3.6	7.1	965	2.9
Europe	United Kingdom	2009	10.9	3.0	6.9	4,245	3.6
Africa	Mauritius	2008	11.8	1.9	6.8	84	6.2
Europe	Macedonia	2003	9.5	4.0	6.8	137	2.4
Europe	Italy	2007	10.0	2.8	6.3	3,757	3.6
Americas	Costa Rica	2009	10.2	1.9	6.1	280	5.4
Americas	Nicaragua	2006	9.0	2.6	5.8	319	3.5
Americas	Panama	2008	9.0	1.9	5.5	186	4.7
Americas	Colombia	2007	7.9	2.0	4.9	2,191	4.0
Americas	Brazil	2008	7.7	2.0	4.8	9,206	3.9
Europe	Uzbekistan	2005	7.0	2.3	4.7	1,221	3.0
Africa	Seychelles	2008	8.9	0.0	4.6	4	N/A
Europe	Cyprus	2008	7.4	1.7	4.5	36	4.4
Europe	Georgia	2009	7.1	1.7	4.3	188	4.2
Europe	Israel	2007	7.0	1.5	4.3	306	4.7
Americas	Mexico	2008	7.0	1.5	4.2	4,565	4.7
Europe	Albania	2003	4.7	3.3	4.0	124	1.4
Eastern Medit.	Bahrain	2006	4.0	3.5	3.8	28	1.1
Americas	Belize	2008	6.6	0.7	3.7	11	9.4
Americas	St.Vincent & Grenadines	2008	5.4	1.9	3.7	4	2.8
Americas	Guatemala	2008	5.6	1.7	3.6	492	3.3
Americas	Paraguay	2008	5.1	2.0	3.6	222	2.6
Europe	Greece	2009	6.0	1.0	3.5	391	6.0
Americas	Barbados	2006	7.3	0.0	3.5	9	N/A
Europe	Malta	2008	5.9	1.0	3.4	14	5.9
Americas	Venezuela	2007	5.3	1.2	3.2	896	4.4
Europe	Tajikistan	2001	2.9	2.3	2.6	163	1.3
Americas	Saint Lucia	2005	4.9	0.0	2.4	4	N/A
Americas	Dominican Republic	2005	3.9	0.7	2.3	220	5.6
Western Pacific	Philippines	1993	2.5	1.7	2.1	848	1.5
Europe	Armenia	2008	2.8	1.1	1.9	63	2.5
Eastern Medit.	Kuwait	2009	1.9	1.7	1.8	62	1.1
Americas	Peru	2007	1.9	1.0	1.4	407	1.9
Americas	Bahamas	2005	1.9	0.6	1.2	4	3.2
Africa	South Africa	2007	1.4	0.4	0.9	420	3.5
Africa	Sao Tome and Principe	1987	0.0	1.8	0.9	1	0.0
Europe	Azerbaijan	2007	1.0	0.3	0.6	55	3.3
SE Asia	Maldives	2005	0.7	0.0	0.3	1	N/A
Eastern Medit.	Iran	1991	0.3	0.1	0.2	111	3.0
Eastern Medit.	Egypt	2009	0.1	0.0	0.1	52	N/A
Eastern Medit.	Jordan	2008	0.2	0.0	0.1	6	N/A
Americas	Jamaica	1990	0.3	0.0	0.1	3	N/A
Eastern Medit.	Syria	1985	0.2	0.0	0.1	10	N/A
Americas	Grenada	2008	0.0	0.0	0.0	0	N/A
Americas	Haiti	2003	0.0	0.0	0.0	2	N/A
Americas	Antigua and Barbuda	1995	0.0	0.0	0.0	0	N/A
Americas	Saint Kitts and Nevis	1995	0.0	0.0	0.0	0	N/A
Americas	Honduras	1978	0.0	0.0	0.0	0	N/A

## 4. Discussion

The WHO has measured suicide since 1950—shortly after its creation in 1948. The list of countries, which have been leading in suicide rate in the world, is not a long one. Japan had the highest rate in the 1950s, Hungary was the top country for the next three decades, and Lithuania took over in the early 1990s for another two decades.

Some other countries also deserve to be mentioned. Although available data are much scarcer before the 1950s, it is plausible to believe that Switzerland has been a world leader in suicide rate because coming into the 1950s its rate was already declining from higher levels in 1930s and 1940s [13]. Measurement of suicide rate has usually been more difficult in developing economies with less resources. Differently from the WHO figures, it has been estimated that the suicide rate in Sri Lanka has reached 47 cases per 100,000 inhabitants in the first half of 1990s [14] which would make it briefly the leading country between the ‘Hungarian period’ and the ‘Lithuanian period’.

Suicide has been a big issue in Finland and Denmark but even when the rates surpassed 30 per 100,000 inhabitants, they were dwarfed by more extreme rates in Hungary at the same time. Similarly, very high suicide rates in Russia, Latvia, and Estonia were overshadowed by those of Lithuania. In the American region highest rates have been observed in Cuba and more recently in Guyana, which have experienced rates above 20 and 25 per 100,000 inhabitants respectively.

Generally the suicide trend has been downward in Europe and there are currently no Western European welfare states in the world top ten for suicide rates. However, a different development has been evident in some other parts of the world—most notably in South Korea where a huge increase has occurred over the last 10 years. The suicide rate now exceeds 30 cases per 100,000 inhabitants and unless there is a dramatic turnaround of the trend, South Korea will take over the world leader’s position from Lithuania in a few years. Efforts have been made to explain the South Korean phenomenon, Kwon has suggested that the current situation has evolved due to the Asian economic crisis of 1997/98 and population cohort effects [15].

Ranking countries according to suicide rates based on data about different years is not perfect, although usually differences are not large. Near the top of the list in Table 3 it should be noted that according to several authors the suicide rate in Sri Lanka has been falling from 47 in 1995 to 24 per 100,000 inhabitants in 2005 [16,17]

The biggest contributors to the absolute number of suicides in the world are China and India. According to Vijayakumar in 2004 more than half the suicides (54%) in the world occur in China and India [18]. In both of these countries, however, we cannot be sure of the accuracy of the suicide numbers provided to the WHO. Law and Liu concluded in their publication in 2008 that to date, China does not have a comprehensive vital statistics reporting system; there exists a relatively wide range of figures on suicide rates [19]. Phillips reported in 2002 [20] that the official figure of 13.9 suicides per 100,000 inhabitants for the period 1995–1999 was an understatement and offered his own estimated suicide rate of 23 per 100,000. This is partially because the official mortality figures provided to the WHO are based on data from about 10% of the population, although urban and rural sample areas are calculated separately. For India nationally representative cause of death distributions were derived from detailed tabulations from the Million Deaths Study and adjusted to the 2008 all-cause envelope [12].

One million suicide deaths in the world per year is a figure often used in discussions and presentations about the magnitude of the phenomenon. The latest WHO estimate of 782,000 suicides in a year is a notably smaller amount. However, this includes neither injury deaths of undetermined intent nor deaths from unknown causes which are generally believed to hide some suicides. Considering this underreporting one million suicide deaths per year is a worthwhile estimate.

According to presented estimates there is a fourfold regional difference in the world in the male-female rate ratio of suicides. Europe, especially Eastern Europe, had the highest ratios while Asian countries had the lowest. China is believed to be the only country where females have a higher suicide rate than males. Cultural factors and regional differences in socio-economic situation play an important role in this. There will be changes in this indicator over time as cultural norms shift and economies take different development paths.

## 5. Limitations

An undeniable limitation of this kind of world overview is the unavailability of data for many countries—therefore estimates have been used where necessary. Some countries have not updated their reported data to the WHO during the last years, so for a dozen countries latest data are from before the year 2000. Suicide registration procedures differ across countries making the direct comparison less trustworthy. Deaths of undetermined intent have been omitted from this analysis due to lack of worldwide data although in some countries they mask a notable part of other categories of injury deaths, completed suicides among them.

## 6. Conclusions

The WHO has made valiant efforts to improve the reporting of injury mortality and as a result data about suicide from over 100 countries are now available as well as current estimates for the whole world. Analysis shows that during the last 50 years the heart of the problem of suicide mortality has shifted from Western Europe to Eastern Europe and now seems to be shifting to Asia. China and India are the biggest contributors to the number of suicides in the world, while South Korea has experienced enormous growth of the suicide rate during the past decade.
